# The prognostic and protective roles of heart-rate variability in glioblastoma: making GBM less vague

**DOI:** 10.1007/s11060-025-05010-3

**Published:** 2025-06-12

**Authors:** Yori Gidron, Niv Katan, Nour Mansour, Rachel Grossman

**Affiliations:** 1 Faculty of Social Welfare and Sciences, University of Haifa, Haifa, Israel; 2 Department of Neurosurgery, Rappaport Faculty of Medicine, Technion - Israel Institute of Technology, Rambam Health Care Campus, Haifa, Israel

**Keywords:** Glioblastoma, Heart rate variability, Prognosis, Vagal activity

## Abstract

**Purpose:**

Recent data has shown the role of vagal nerve activity in cancer prognosis. This study examined the prognostic value of vagal nerve activity in patients with newly diagnosed glioblastoma.

**Methods:**

Eighty-eight patients underwent a surgical resection or biopsy of glioblastoma in a single institution between 2003 and 2023. Inclusion criteria were: (1) age over 18; (2) diagnosed with newly diagnosed glioblastoma; (3) having an ECG prior to surgery. The predictor was vagal nerve activity indexed by heart rate variability (HRV) and obtained retroactively from 10 s ECGs. The main endpoint was overall survival (OS).

**Results:**

HRV significantly predicted OS independent of confounders such as age, performance status, or extent of tumor resection (EOR), but only among younger patients (≤ 65 years old). Patients with low and high HRV had OS of 13.2 and 20.2 months, respectively (p < 0.05). We found evidence for a moderating effect of HRV in relation to other prognostic factors. Specifically, EOR predicted death only in patients with low HRV, but not with high HRV. Similarly, KPS tended to predict death only in patients with low, but not high HRV.

**Conclusions:**

In this study, we have shown for the first time the clinical and prognostic value of vagal nerve activity indexed by measurement of HRV in patients with newly diagnosed glioblastoma. If replicated in prospective studies, vagal nerve activity may have the potential to be a new therapeutic target in newly diagnosed glioblastoma.

## Introduction

Glioblastoma is the most common type of primary malignant brain tumors [1, 2]. Despite advances in treatment modalities, it remains largely incurable, and its prognosis remains grim with a median survival time of 12–14 months from diagnosis [1, 3]. Multiple clinical factors are known to affect overall survival (OS), including age, preoperative and postoperative Karnofsky Performance Status (KPS), extent of resection (EOR), administration of adjuvant therapy and promoter methylation status of O^6^-methylguanine-DNA methyltransferase (MGMT) [1, 4–6]. Searching for unique strategies to improve the prognosis of patients with glioblastoma emphasizes the need to identify new clinical factors with protective impact, which can predict a better clinical outcome. Furthermore, certain protective factors may possibly weaken (statistically moderate) the adverse effects of other prognostic factors.

A potential candidate to meet these tasks is the vagal nerve, whose activity is indexed by heart-rate variability (HRV), the fluctuations in the intervals between normal heartbeats [7]. Heart rate variability is considered an important measure of autonomic nervous system activity, especially of the parasympathetic branch. A large number of studies has demonstrated the prognostic significance and protective role of HRV in many pathological conditions such as cardiovascular disease [8, 9], Alzheimer’s disease [10], pancreatitis [11] and cancer [12–22]. Significant positive correlations between HRV and survival time have been reported in patients with terminal hepatocellular carcinoma [14, 16], diffused large B-cell lymphoma [12], pancreatic cancer [18], patients with non-small cell lung cancer [17, 23], cancer with brain metastases [24] and breast cancer [21, 25], independent of other well-known prognostic factors. These results were confirmed in two systematic reviews [11, 26] and in one meta-analysis [27]. Importantly, some studies found that higher HRV weakened the prognostic role of other factors (statistical moderation). For example, Atar et el. [12] found that relapse of patients with diffused large B-cell lymphoma predicted cancer mortality, but only when patients had low HRV. The protective role of the vagus in cancer is thought to be partly due to its anti-inflammatory effects [28, 29], and this has been shown in pancreatic cancer, where lower C-reactive protein (CRP) significantly mediated the relations between HRV and longer survival [18]. Inflammation also has been shown to have a prognostic role in glioblastoma [30]. However, to the best of our knowledge, no study has assessed the prognostic role of HRV in patients with primary brain cancer. Based on the converging evidence reviewed above, we hypothesized that higher HRV would predict better OS in glioblastoma patients. Furthermore, based on previous data, these results may be even stronger among patients younger than 65 years old [19]. Finally, we also examined whether HRV moderated the relation between other well-known prognostic factors such as KPS and EOR with OS.

## Methods

We conducted a retrospective analysis (formally a historical prospective design) to evaluate the prognostic value of HRV among patients with newly diagnosed glioblastoma in a single neurosurgical center between 2013 and 2023. The study was approved by the institutional review board (approval 0425-23). Given its retrospective nature, no informed consent was required. Overall, a total of 88 adult patients (age > 18 years) with newly diagnosed glioblastoma who had full data set, comprised the study group.

### Clinical characteristics

The medical records of the study patients were reviewed to collect data on demographics, patients’ age, gender, hand dominance, comorbidities, body mass index (BMI), neutrophil to lymphocytes ratio (NLR), CRP, tumor side, tumor location, tumor multifocality and presenting symptoms. The KPS was used to assess preoperative and postoperative functional status. Additional parameters included pre-operative steroids consumption, operative course, EOR, post-operative complications, postoperative neurological status and post operative oncological treatment. Pathology was determined according to the World Health Organization (WHO) criteria [2].

### Measurement of vagal activity

This was derived from patients’ brief 10-s ECG and was based on the R-R intervals. We manually calculated the standard deviation of normal to normal (SDNN) R-R intervals and the route means square of successive differences (RMSSD) of R-R intervals. Previous studies showed high correlations between HRV derived from ultra-short 10-s ECG and 5-min ECG [31]. Such ultra-short measures of HRV have predicted prognosis in several cancers (e.g., 25).

### Clinical outcomes

This was indexed by overall survival (OS) which reflected both vital status at follow-up and time from diagnosis until death (for deceased patients) or time from study entry until censorship date (1.4.2024 for alive patients).

### Statistical analysis

Values are presented as the mean ± standard deviation for continuous data and as percentages for categorical data. A p-value < 0.05 (two-sided) was considered statistically significant. We examined the univariate relation between each confounder and OS with Cox regression, considering vital status and time to event as the OS outcome. Then, we entered all significant confounders with HRV in the multivariate Cox regression in relation to OS. To preliminarily test the moderating role of HRV, we finally tested relations between significant clinical prognostic variables with death, using Spearman correlations, separately in patients with low and high HRV.

## Results

Our cohort of patients included 88 patients who underwent a surgical resection or biopsy of newly diagnosed glioblastoma in a single institution between 2003 and 2023 and had full dataset. The mean follow-up period was 13.29 ± 11 mo. The patients’ clinical and demographic characteristics are summarized in Table 1 which depicts the means and standard deviation (SD) of the continuous variables and the percentages of cases for categorical variables. The mean age of all patients was 65.08 ± 11.19 yr, with a slight male predominance. Thirty-nine patients were 65 years or younger and 49 patients were older than 65 years. Most of the patients (61%) had a KPS score of > 70 prior to surgery. Most of the patients had a comorbid diagnosis, including hypertension, endocrinopathies, other malignancies, or cardiac or pulmonary diseases. Among patients who were ≤ 65 y/o, 17 patients had no co-morbidities while among patients who were > 65 y/o, only 4 had no co-morbidities (X2(1) = 15.00, p < 0.001). The most common tumor location was the frontal lobe, followed by the parietal lobe. Half of the patients had their tumor located in the left hemisphere, and 9% of the patients had a bilateral tumor involvement. Thirty-five patients underwent biopsy of the tumor and 53 patients underwent surgical resection. The mean EOR for patients who underwent resection of their tumors was 90.96 ± 13.5. Sixty-four patients received at least 4 mg of dexamethasone per day and 22 patients did not receive steroids. These data were not available for 2 patients. The mean pre-operative consumption of daily steroids was 10.5 ± 8.1 mg per day. Patients’ mean SDNN was below the healthy mean of 50 ms.

**Table 1:** Categorical variables

Variables	%
a	
Gender: women, men	41.1, 56.7
Side: left, right, bilateral	51.1, 37.8, 8.9

Variables	Mean ± SD
b	
Age (years)	65.08 ± 11.19
KPS	69.20 ± 12.97
EOR (%)	54.37 ± 46.05
HRV (SDNN; ms)	34.53 ± 39.83
Overall Survival (days)	433.97 ± 394.42

*SD* standard deviation, *SDNN* standard deviation of normal-normal intervals, *HRV* heart rate variability, *KPS* Karnofsky performance scale, *EOR* extent of resection

### Predictors of overall survival

**Fig. 1:**
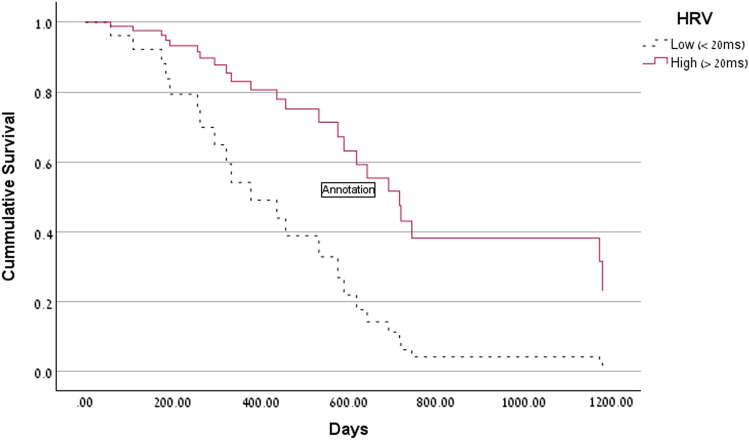
Survival of patients with low versus high heart rate variability (HRV) in patients below age 65

A univariate analysis assessed the relation between demographic and clinical variables and OS, using a Cox Regression. Gender, tumor side and WBC did not predict OS. However, age, KPS and EOR (%) significantly predicted OS. Categorical SDNN tended to significantly predict OS (p = 0.064). In a multivariate Cox regression analysis (Table 2), the only factor which remained significantly and independently associated with improved OS was EOR. Age, KPS, as well as SDNN, were no longer significantly associated with OS. Since HRV declines with age and because previous data on non-small cell lung cancer found that HRV significantly predicted survival only in relatively younger patients [19], we defined the age cutoff at 65 years, near the median age of our sample. As shown in Table 3, only in younger patients (≤ 65 years old), did high SDNN significantly predict better OS (Table 3a), independent of age, KPS and EOR, but HRV did not predict OS in relatively older patients (> 65 years old) (Table 3b). These results are depicted graphically in Fig. 1. Receiving dexamethasone tended to predicted mortality (by univariate logistic regression: p < 0.07) and tended to be significantly more prevalent (90.5%) in the alive patients than in the deceased patients (69.2%). Importantly, steroids did not moderate the relation between HRV and survival – means, HRV did not predict OS in patients who receive or did not receive steroids (both ps > 0.05).

**Table 2:** Multivariate Cox regression of confounders and heart-rate variability (SDNN) in relation to overall survival

Variable	B	SE	Sig	R.R	Lower R.R	Upper R.R
KPS	−0.02	0.11	0.10	0.98	0.96	1.00
EOR	−0.01	0.00	0.00	0.99	0.98	0.99
Age	0.02	0.01	0.16	1.02	0.99	1.04
SDNN	−0.39	0.27	0.14	0.68	0.40	1.14

*SE* standard error, *Sig.* statistical significance, *R.R* relative risk, *Lower/Upper R.R* lower and upper 95% confidence interval, *KPS* Karnofsky performance status, *EOR* extent of resection, *SDNN* standard deviation of normal-normal intervals

**Table 3:** Multivariate Cox regression, of confounders and heart-rate variability (SDNN) in relation to overall survival, in younger and older patients

Variable	B	SE	Sig	R.R	Lower R.R	Upper R.R
a. Patients’ age ≤ 65						
KPS	−0.04	0.02	0.08	0.96	0.92	1.00
EOR	−0.01	0.01	0.23	0.99	0.98	1.00
Age	0.01	0.03	0.63	1.01	0.96	1.07
SDNN	−1.19	0.53	0.02	0.30	0.11	0.85
b. Patients’ age > 65						
KPS	−0.02	0.01	0.13	0.98	0.96	1.01
EOR	−0.02	0.00	0.00	0.98	0.97	0.99
Age	−0.03	0.03	0.44	0.97	0.91	1.04
SDNN	−0.08	0.32	0.79	0.92	0.49	1.73

*SE* standard error, *Sig.* statistical significance, *R.R* relative risk, *Lower/Upper R.R* lower and upper 95% confidence interval, *KPS* Karnofsky performance scale, *EOR* extent of resection, *SDNN* standard deviation of normal-normal intervals

### Does high HRV moderate the prognostic roles of other predictors?

Given that previous studies showed that high vagal nerve activity, indexed by HRV, weakened (moderated) the prognostic effects of other known predictors such as of cancer relapse [12], we examined this phenomenon in the present study as well. We examined whether age, KPS and EOR, which univariately and significantly predicted death in the full sample, also did so in patients with low versus high HRV. We used a cut-off of median SDNN of 20 ms, reported in several cancers [17]. We used Spearman correlations (see Table 4). In patients with low HRV, age and EOR significantly predicted survival, and KPS tended to significantly predict survival. In contrast, in patients with high HRV, only age significantly predicted survival. Therefore, high HRV moderated the prognostic role of EOR and tended to do so for KPS as well, in relation to predicting death.

**Table 4:** Spearman correlations between confounders and death, as function of patients’ heart rate variability (HRV)

Variable	Death	
	Low HRV	High HRV
Age	0.33*	0.33*
KPS	−0.27!	−0.09
EOR	−0.48***	0.02

*HRV* heart-rate variability, *EOR* extent of resection, *KPS* Karnofsky performance scale. ! p < 0.10; * p < 0.05; ** p < 0.001

## Discussion

In this study we have shown for the first time the clinical and prognostic value of vagal nerve activity indexed by measurement of HRV in patients with newly diagnosed glioblastoma. In the entire cohort of patients, HRV tended to predict OS, but did not reach statistical significance. However, in younger patients (< 65 years), high SDNN significantly predicted better OS, independent of confounders such as age, performance status and EOR. A similar finding was also reported in a previous publication assessing the prognostic role of HRV in patients harboring NSCLC [19]. Among 133 patients with NSCLC, HRV did not predict OS and survival time in the entire cohort, but positively predicted survival time in patients under the age of 65, independent of other confounders [19].

One possible explanation to the linkage between HRV and OS in younger patients may be related to the ability of the vagus nerve to inhibit inflammation [28, 29]. It has already been reported that systemic inflammation is associated with shortened OS in patients with various type of malignancies [32]. Fibrinogen is considered a biomarker in the regulation of inflammation, tumor migration, and angiogenesis [33]. High levels of fibrinogen in the plasma are also associated with shorter OS in a variety of tumors [34, 35]. C-reactive protein (CRP) is a non-specific acute-phase protein that is synthetized in the liver. Several studies have suggested that increased serum levels of CRP are associated with poor outcomes. CRP was also found to be an inflammation-related biomarker that can predict survival in GBM patients [30]. Indeed, both CRP and fibrinogen, markers of inflammation, were found to predict OS in patients with glioblastoma [36]. Older people often have several diseases, many of which are related to inflammation (e.g., heart disease, diabetes). It is plausible that the cholinergic anti-inflammatory pathway may be more effective and less disrupted in younger patients with fewer co-morbidities, and this may explain why HRV predicted better OS in patients younger than age 65.

In addition, we found evidence for a moderating effect of HRV in relation to other prognostic factors. Specifically, EOR significantly predicted death only in patients with low HRV, but not in patients with high HRV. Similarly, KPS tended to significantly predict death only in patients with low but not high HRV. These results echo those of Atar et al. [12] who found that only when HRV was low, did cancer relapse predict mortality, while this did not occur in patients with high HRV. Our observations also echo those of another study which found that cancer-stage predicted increases in tumor marker levels in prostate and colorectal cancer, but again only in those with low, but not high HRV [14].

The fact that EOR predicted better OS only in patients with low HRV may suggest that higher HRV could possibly "override" at times certain important prognostic factors such as EOR in relation to patients’ survival. This pattern of results supports the hypothesized protective role of the vagal nerve in cancer [26]. While this echoes past studies [12], this finding needs to be replicated in future studies with larger samples.

## Limitations

This study had several limitations. It was a retrospective study, and as such, we could not consider other important confounders [37]. It has been already shown that HRV may be altered by various factors such as duration and time of measurement, breathing, drugs, and other confounding factors which may alter each metric in different ways and could not be added to the current analysis [38]. Other factors that may affect HRV and were only partially available in this analysis include health status, food, water consumption and bladder filling, as well as genetics, body mass index, coffee consumption, smoking, drugs, chronic diseases, or fatigue. To limit these factors’ influences on HRV, some authors recommend to take measurements in supine position early in the morning [39]. Drugs such as Alpha-1 and Beta blockers, Benzodiazepines, Bupropion, Clozapine, Cocaine, Fanatrex, Flecainide, Scopolamine, Thioridazine, Tricyclics can potentially lead to the highest variability in HRV in mid age patients [40]. We used a brief 10 s ECG from which we derived the index of HRV and we did not measure more precise indexes of inflammation such as CRP or pro-inflammatory cytokines. An additional limitation of this retrospective study is the inability to assess parameters which impact both HRV and possibly prognosis such as mental distress and physical fitness. Other valuable parameters that was not available in this retrospective analysis included post-operative HRV and inflammatory parameters such as CRP that were available to only 24 patients in the current cohort, thus we decided not to include it in our analysis.

Future studies should replicate these results using a prospective study and a larger sample, a longer measure of HRV and more specific measures of inflammation. Nevertheless, these results propose that vagal nerve activity may have a prognostic value in younger patients with glioblastoma and may even override the prognostic value of other predictors (KPS, EOR) in patients with glioblastoma. These results are in line with a growing line of research showing the prognostic role of vagal nerve activity as indexed by HRV in various types of cancer [11, 26]. Following replication of these findings while addressing our limitations, future intervention studies could then test the effects of vagal nerve activation on the prognosis of patients with glioblastoma.

## Data Availability

No datasets were generated or analysed during the current study.

## Abbreviations

SDDN: Standard Deviation of all Normal to Normal intervals
HRV: Heart rate variability
EOR: Extent of resection
OS: Overall survival
RMSSD: Route means square of successive differences
MGMT: O^6^-methylguanine-DNA methyltransferase
KPS: Karnofsky Performance Scale
CRP: C-reactive protein
NLR: Neutrophil to lymphocytes ratio
WHO: World Health Organization
SD: Standard deviation
NSCLC: Non-small cell lung cancer
